# Supplementary material for: Peptide vaccine-treated, long-term surviving cancer patients harbor self-renewing tumor-specific CD8+ T cells
Source: Nat Commun. 2022 Jun 3;13:3123. doi: 10.1038/s41467-022-30861-z (PMC9166698; doi:10.1038/s41467-022-30861-z)
Supplement: Supplementary file 1 — Supplementary Information [file 41467_2022_30861_MOESM1_ESM.pdf]

Peptide vaccine-treated, long-term surviving cancer patients harbor self-renewing tumor-specific $CD8^+$ T cells

Mizukoshi et al.

Supplementary Figure 1

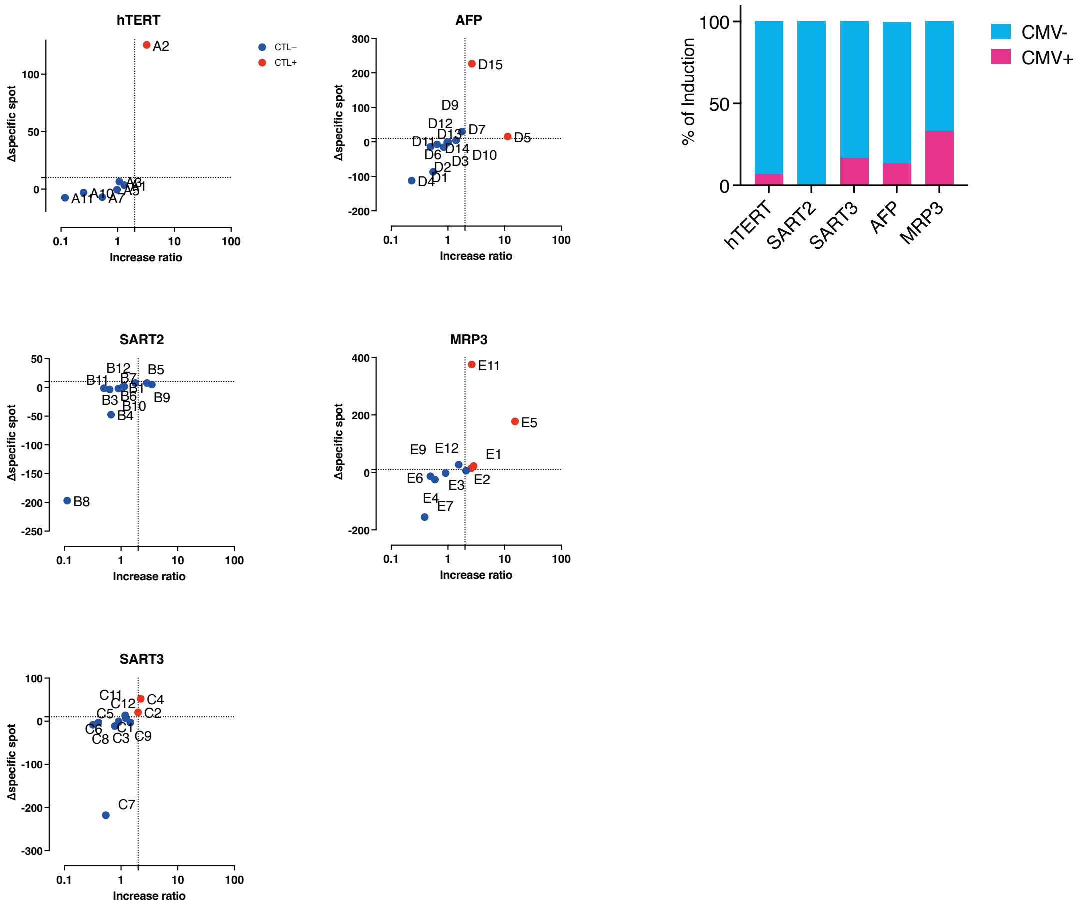

Supplementary Figure 1

Induction of cytomegalovirus (CMV)-specific immune responses after peptide vaccine treatments. PBMCs were collected before and at 3–6 months after vaccination (pre and post, respectively) from each participant and tested to assess CMV-specific IFN-γ production by an ELISpot assay following the manufacturer's instructions. Briefly, pre-PBMCs or post-PBMCs were seeded onto capture antibody-coated PVDF membrane plates with or without $CMVpp65_{328}$ peptide. The plates were incubated overnight, secreted IFN-γ was captured by the detection antibodies, and spots were developed by HRP. Patients from the five clinical trials were tested. Immune responses measured by the IFN-γ ELISpot assay are plotted in X-Y graphs with annotations of the patient IDs. The X-axis represents the fold change of specific spots and the Y-axis indicates an increase of the specific spot number by comparing pre and post samples. Because positive induction of CMV-specific T lymphocytes was defined as both a more than 10 increase and more than two-fold increase of the specific spot number, dotted lines are drawn at X=2 and Y=10 to distinguish cases with positive induction of CMV-specific responses. Positive inductions are highlighted in red and negatives are highlighted in blue. If both pre and post were 0, X was defined as 1. If only pre was 0, the X-value was offset out of X=100 and if only post was 0, the X-value was placed next to X=0.1. The proportions of positive induction in each peptide study are shown in bar graphs. Positive induction of CMV-specific responses is filled in magenta and negative induction is shown in cyan.

Supplementary Figure 2

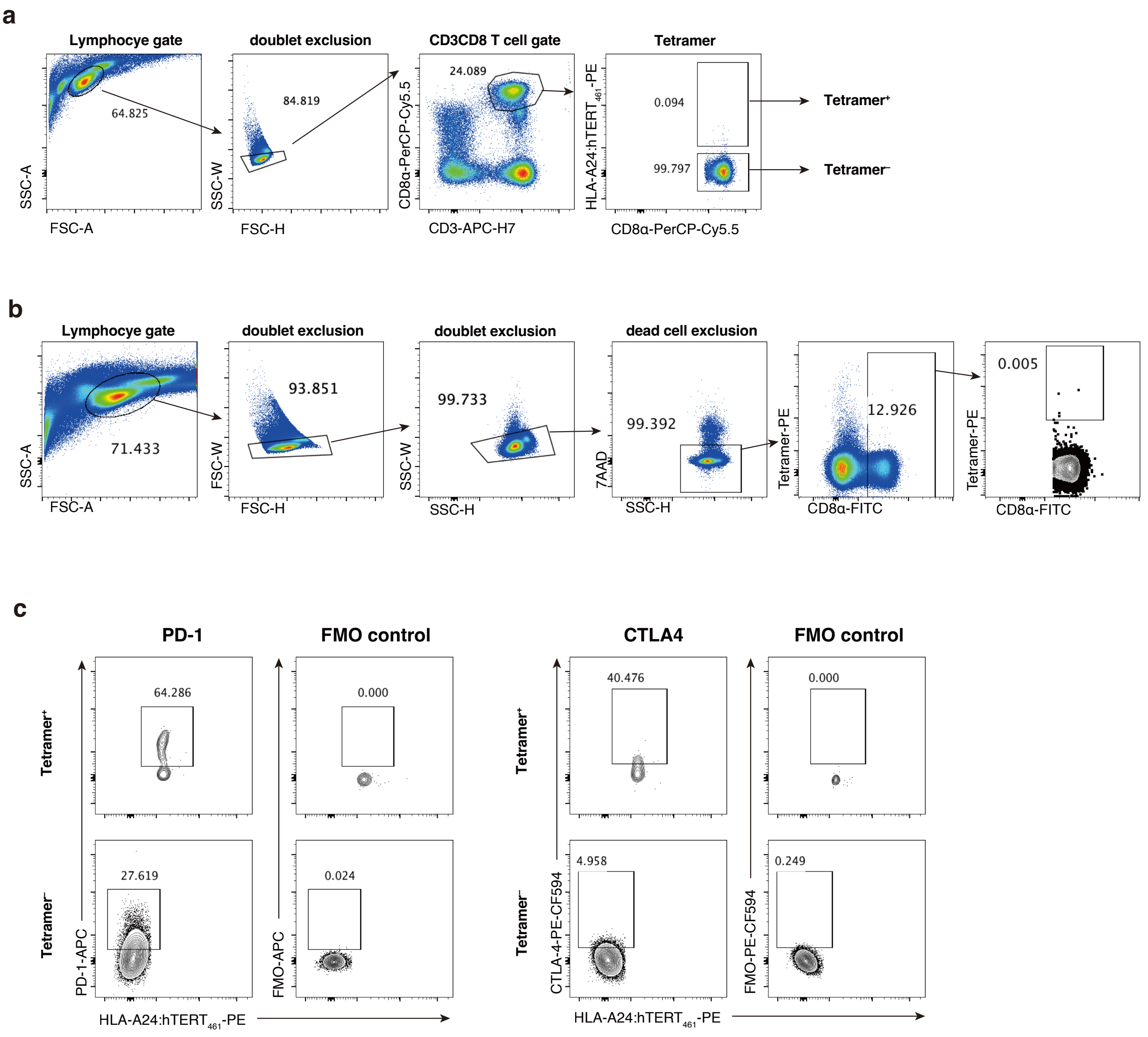

Supplementary Figure 2

Gating strategy on flow cytometry. We took two types of strategies; one is for analysis **(a)** and the other is for sorting **(b)**. For analysis, we identified CD8+ T cells gated on FSC/SSC gate-in, single-cell gate-in, and CD3e+CD8a+. For frequency comparison, we used the same gating sets on FlowJo and obtained the results as shown in Fig. 3. For sorting, to avoid antibody-related effects during incubation on transcriptome profiles we did not use anti-CD3 antibody. Instead, we adopted 7AAD to exclude dead cells and strict doublet exclusion gates. We validated PD-1 staining and CTLA-4 staining within tetramer+ and tetramer− fractions in **Fig. 3g** using fluorescence minus one (FMO) controls. A7 PBMC sample was used in this experiment. Reproducibility of the staining patterns and frequencies was verified (**c**).

Supplementary Figure 3

Design of sorting strategy

**A2 lymphocytes (7AAD⁻ lymphocyte gate)**	**30,000 cells**					
**A2 tetramer+ cells**	**1,000 cells**	**Combined**	➡	**20,000 cells**	➡	**cDNA library preparation**
**D3 tetramer+ cells**	**1,000 cells**					

Sort results

Time point	A2 lymphocyte	A2 tetramer+	D3 tetramer+
1-year	30,000	500	500
5-year	30,000	1,100	983

Supplementary Figure 3

Sorting strategy for single-cell RNAseq analyses. We strategized single-cell RNAseq library preparation to overcome the low frequency of tetramer^{+} T cells. A2 HLA-A24:hTERT$_{461}$ tetramer^{+} cells were sorted out (1,000 cells) and combined with A2 lymphocytes (30,000) that were sorted from the 7AAD^{-} lymphocyte gate. Sorted HLA-A24:AFP$_{357}$ tetramer^{+} cells from D3 were added to the cell mixture as well. Twenty thousand of the cell mixture were subject to cDNA library preparation using the Chromium platform (10x Genomics U.S.). Sort results are shown in a table. We prepared 2 libraries from different time points (1-year and 5-year).

Supplementary Figure 4

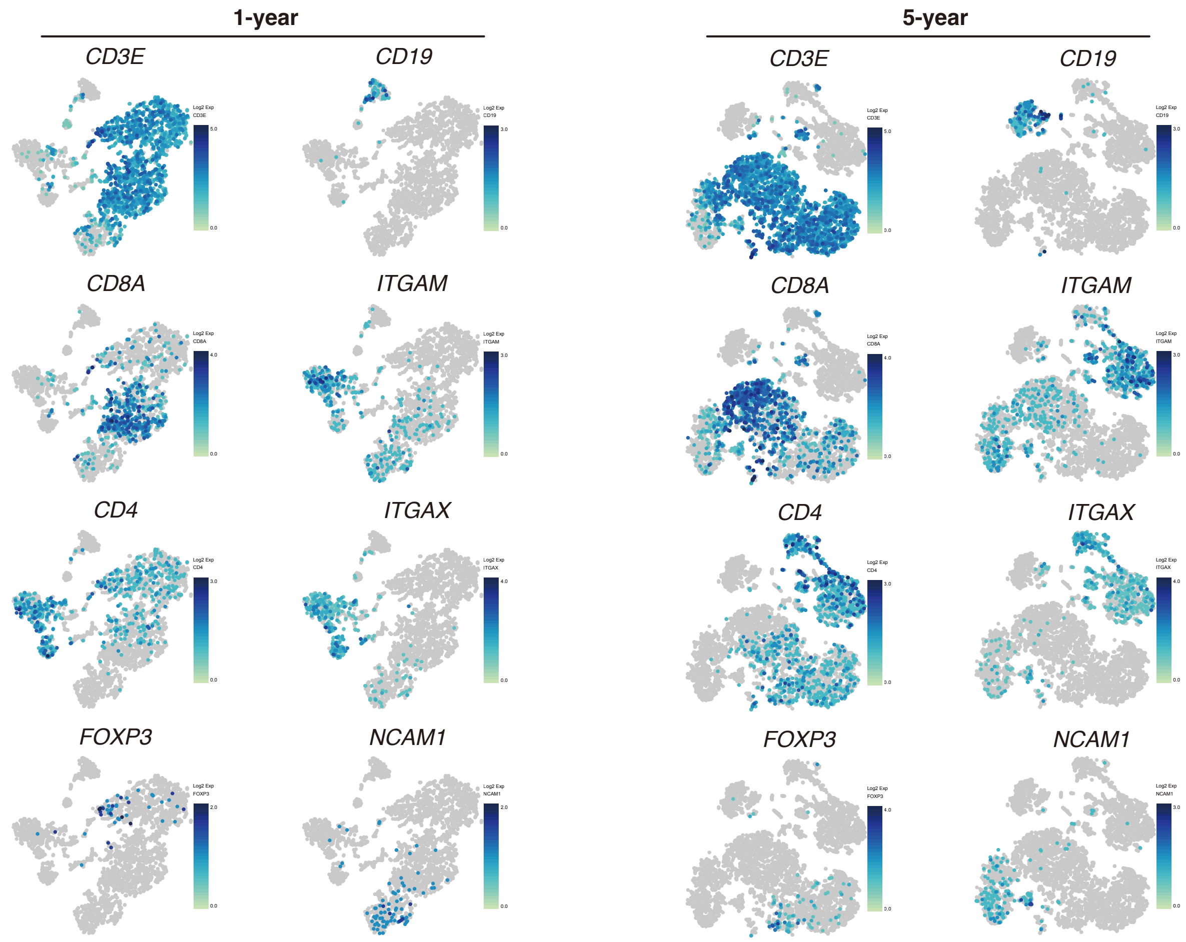

Supplementary Figure 4

Cell populations identified by lineage markers. CD3E, CD8A, CD4, FOXP3, CD19, ITGAM, ITGAX, and NCAM1 expression was visualized on t-SNE plots to identify $CD4^+$ T, $CD8^+$ T, regulatory T, B, $CD11b^+$, $CD11c^+$, and natural killer (NK) cells.

Supplementary Figure 5

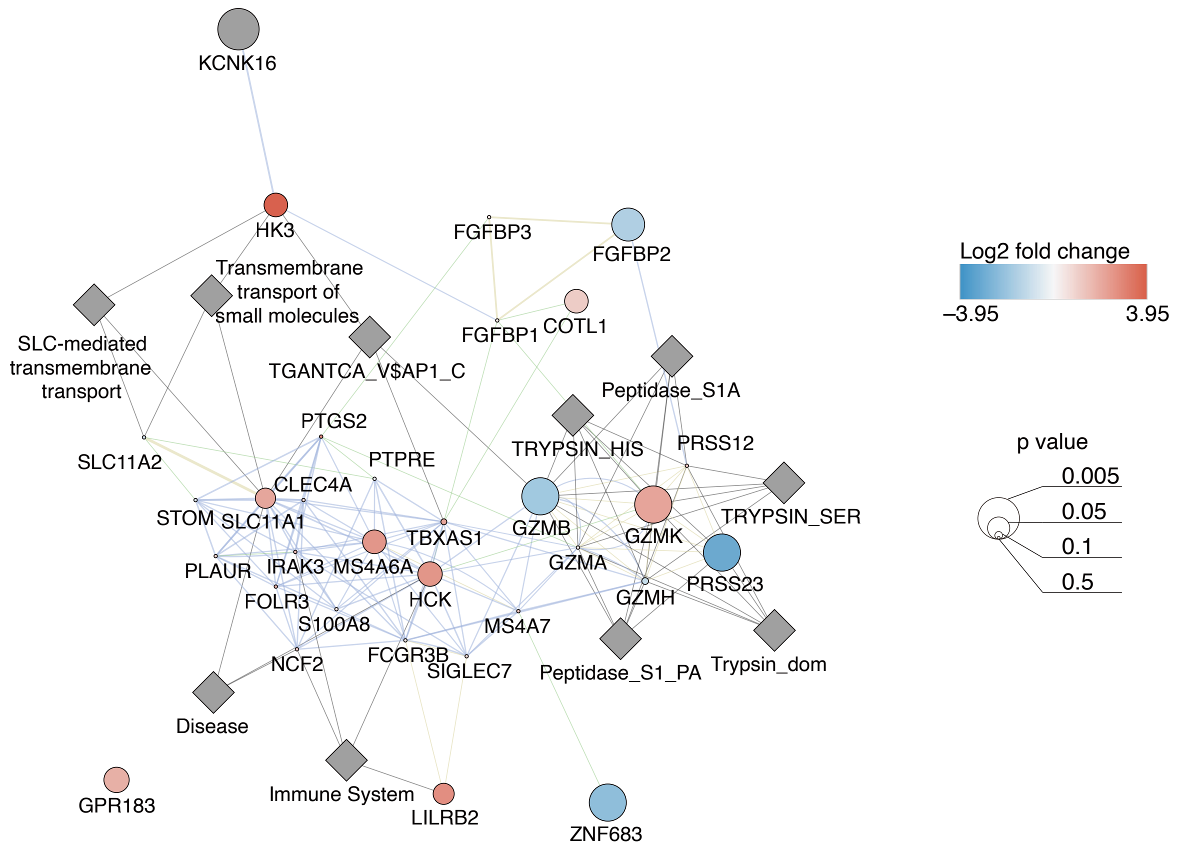

Supplementary Figure 5

Pathway analysis of a dataset of differentially expressed genes in cluster 2 of the 1-year sample compared with those of total CD8+ T cells in the 1-year single-cell RNAseq sample. Genes (circles) and related pathways (diamonds) are connected by edges in accordance with the GeneMANIA biological network prediction database. Upregulated genes are shown in red and downregulated genes are blue. Sizes of the nodes represent P-values of the differences in gene expression (small P value is a large circle and vice versa).

Supplementary Figure 6

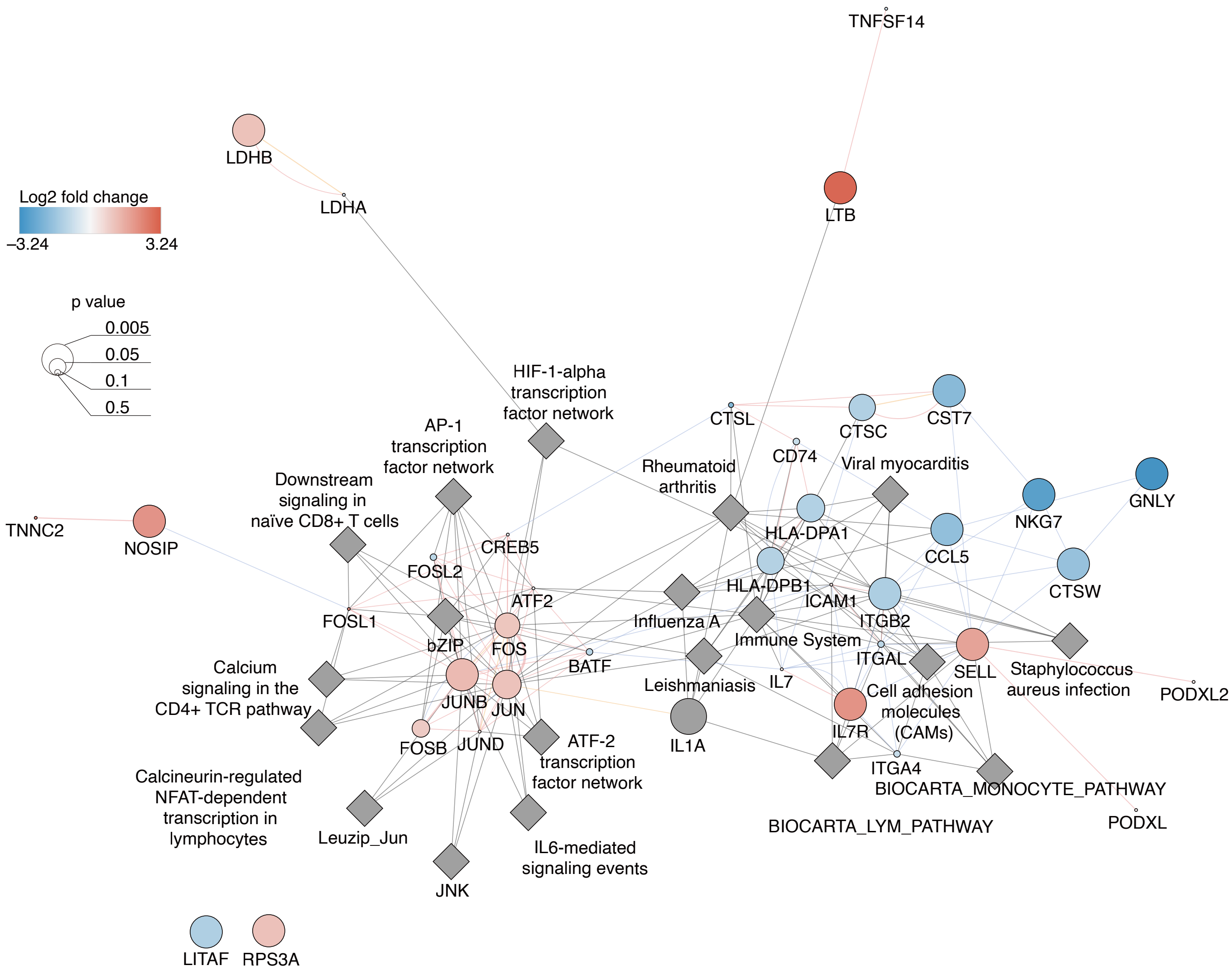

Supplementary Figure 6

Pathway analysis of a dataset of differentially expressed genes in cluster 2 of the 5-year sample compared with those of total CD8⁺ T cells in the 5-year single-cell RNAseq sample. Genes (circles) and related pathways (diamonds) are connected by edges in accordance with the GeneMANIA biological network prediction database. Upregulated genes are shown in red and downregulated genes are blue. Sizes of the nodes represent P-values of the differences in gene expression (small P value is a large circle and vice versa).

Supplementary Figure 7

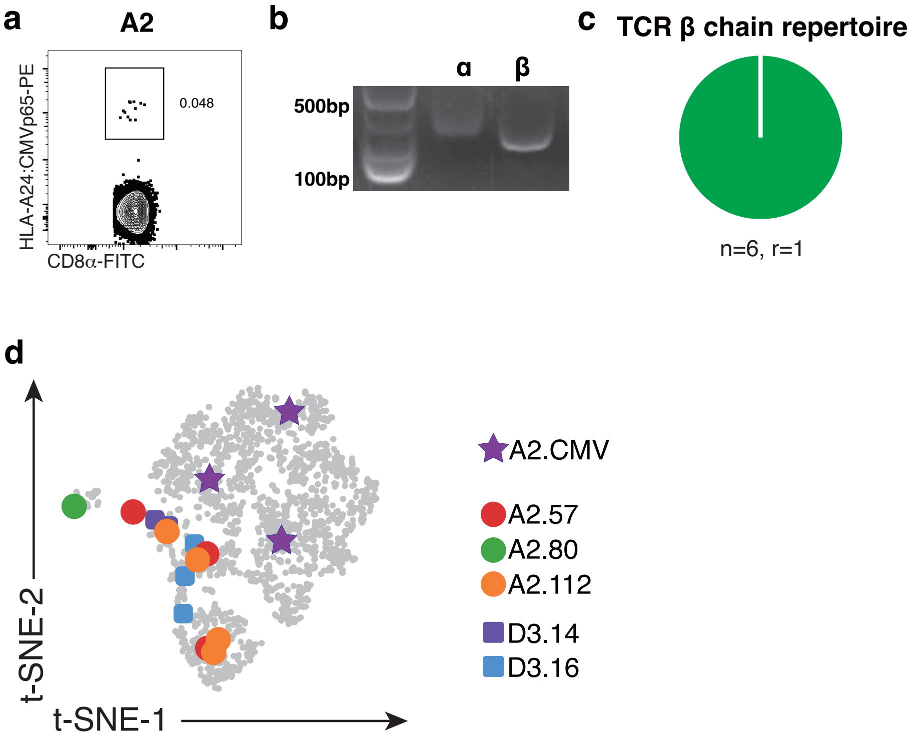

Supplementary Figure 7

Overlay mapping of CMV-specific T cells on the combined t-SNE plot. The enormous VDJ information of single-cell RNAseq lacks specificity knowledge except for hTERT and AFP that we had already analyzed. We added another specificity perception of CMV; a foreign antigen. To know CMV-specific TCR gene sequences, HLA-A24:CMVpp65$_{328}$ tetramer^{+} CD8 T cells were sorted out onto a PCR plate with one cell per well (**a**). Using the hTEC10 technology, RT-PCR amplified TCR αβ chains from the single cells (**b**). Direct sequencing revealed a homogeneous TCR β chain of TRBV9/TRBJ1-1/TRBD1/CDR3: ASSVGQGAYTEAF was shared among the tetramer^{+} cells (n, number of clones; r, number of repertoires, see **Supplementary Table 6**) (**c**). The newly identified CMV-specific TCRβ chain was retrieved in the single-cell RNAseq dataset and found in 3 cells (1 in 1-year and 2 in 5-year). The identified CMV-specific T cells are mapped on the combined CD8 T cell plot and shown in purple stars with the peptide-specific T cells (**d**).

Supplementary Figure 8

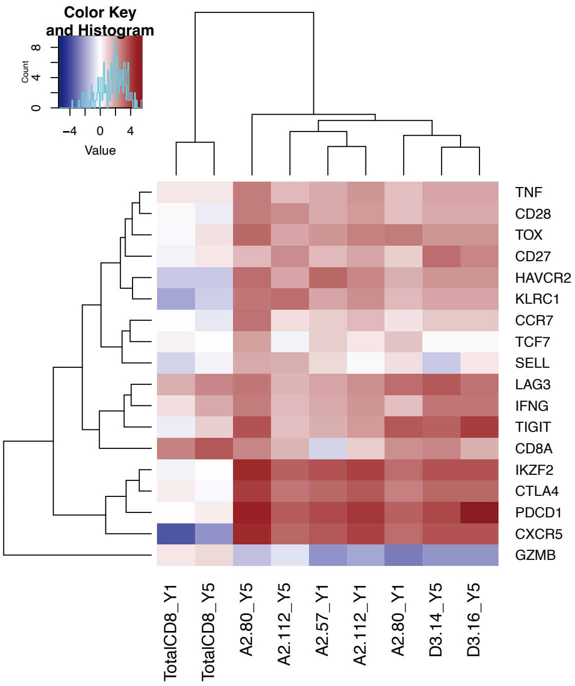

Supplementary Figure 8

Heatmap of gene expression of interest. Log2 fold change values of a series of genes associated with the T cell status were calculated in comparison with total cells of 1- and 5-year samples combined and presented in a heatmap table. Nine subpopulations were found after clustering: CD8+ cells in 1- and 5-year samples (labeled as TotalCD8_Y1 and TotalCD8_Y5, respectively) and the peptide-specific TCR-positive cells (A2.112_Y1, _Y5, A2.80_Y1, _Y5, A2.57_Y1 D3.14_Y5, and D3.16_Y5).

Supplementary Figure 9

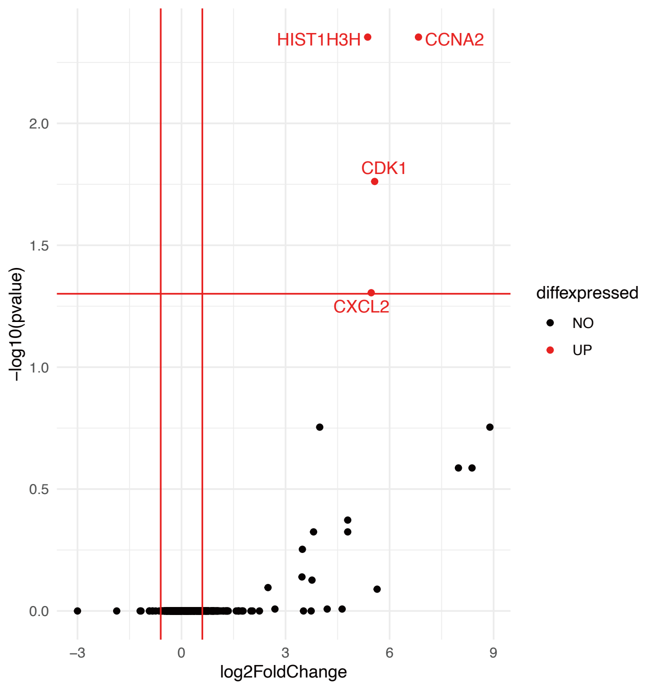

Supplementary Figure 9

Differentially expressed genes identified by comparing peptide-specific T cells (from A2 and D3, combined) with total $CD8^+$ T cells (1- and 5-year samples combined) and displayed as a volcano plot. Red dots and gene symbols in red are significantly upregulated genes as judged by $P<0.05$ and log 2-fold change>1. There were no significantly downregulated genes as judged by $P<0.05$ and log 2-fold change<-1.

Supplementary Table 1

Peptides

Peptide name	Source	Amino acid sequence	Number of specific spots in normal donors (mean + SD)
hTERT$_{461}$	hTERT	VYGFVRACL	0.7+1.2
SART2$_{899}$	SART2	SYTRLFLIL	1.0+1.4
SART3$_{109}$	SART3	VYDYNCHVDL	2.1+1.9
AFP$_{357}$	AFP	EYSRRHPQL	1.8+2.0
AFP$_{403}$	AFP	KYIQESQAL	1.1+1.5
MRP3$_{765}$	MRP3	VYSDADIFL	0.9+1.0
HIV env$_{584}$	HIV env	RYLRDQQLL	1.3+2.0
CMV pp65$_{328}$	CMV pp65	QYDPVAALF	13.3+15.7

Supplementary Table 2

Patients' Demographics and Clinical Characteristics

Characteristic/Group*	A	B	C	D	E
No. of patients	14	12	12	15	12
Age, years					
Median	63	73	70	73	67
Range	52-74	49-79	62-83	55–81	58-74
Male : female ratio	10:4	8:4	9:3	9:6	10:2
Etiology of liver disease					
HCV/HBV/others	9/4/1	7/2/3	7/1/4	10/2/3	7/0/5
Histology of non-tumor liver					
LC/non-LC/ND	12/2/0	5/4/3	5/7/0	13/2/0	10/2/0
Liver function					
Child–Pugh A/B/C	12/2/0	11/1/0	12/0/0	7/8/0	4/8/0
ALT (IU/L)					
Median	33	30	29	46	46
Range	13-157	18-111	22-78	18-137	22-144
AFP (ng/ml)					
Median	24	24	8	2177	135
Range	10-512	10-84	2-183	208–96220	9-14890
BCLC classification					
0/A/B/C/D	7/6/1/0/0	7/5/0/0/0	5/6/1/0/0	0/0/8/7/0	0/0/7/5/0

*: Group A, patients with hTERT-derived peptide vaccine; Group B, patients with SART2-derived peptide vaccine; Group C, patients with SART3-derived peptide vaccine; Group D, patients with AFP-derived peptide vaccine; Group E, patients with MRP3-derived peptide vaccine.

Abbreviation:

AFP, alpha-fetoprotein; Alb, albumin; ALT, alanine aminotransferase; HBV, hepatitis B virus; HCV, hepatitis C virus; LC, liver cirrhosis; PLT, platelet; RFA, radiofrequency ablation; TACE, transcatheter arterial chemo-embolization; T-Bil, total bilirubin; BCLC classification, Barcelona-Clinic Liver Cancer staging classification.

Supplementary Table 3

IFN-γ ELISpot results

Study	Patient ID	hTERT$_{461}$		HIVenv$_{584}$		CMVpp65$_{328}$	
		Pre	Post	Pre	Post	Pre	Post
	A1	0	10.5	2.5	3.5	11.5	15
	A2	0	86.5	0	7	56	181.5
	A3	17	35	0	3	97.5	104
	A4	1	13	0	0	0	0
	A5	0	15	0	0.5	17.5	17
	A6	0	11.5	0	0	0	2
Group A	A7	3.5	13.5	0	0	15	8
hTERT	A8	3.5	15	2.5	0	131	46.5
	A9	2.5	11	5.5	2.5	0	0
	A10	9	6	0.5	0	4	1
	A11	6	4	0	0	8.5	1
	A12	3	5.5	0.5	0	159.5	106
	A13	12.5	14.5	0	0	320.5	212
	A14	16.5	35	0	0	0	0

Study	Patient ID	SART2$_{899}$		HIVenv$_{584}$		CMVpp65$_{328}$	
		Pre	Post	Pre	Post	Pre	Post
	B1	0.5	0	0	1	8.5	9
	B2	0	2	0	5	0	22.5
	B3	0	0	2.5	0	3	1.5
	B4	0	0.5	0	0	143	95.5
	B5	0	0.5	0.5	0	4	11.5
Group B	B6	0	0	5.5	4.5	9.5	17
SART2	B7	0.5	20	5.5	2	19.5	17.5
	B8	0.5	1	12	1	222	25
	B9	0	32	1	2	2	7
	B10	2	14.5	1	1	4.5	5
	B11	1.5	12	1.5	4	9.5	6
	B12	0	0.5	0	0	11	12.5

Study	Patient ID	SART3$_{109}$		HIVenv$_{584}$		CMVpp65$_{328}$	
		Pre	Post	Pre	Post	Pre	Post
	C1	0.5	14	1	4	5.5	8
	C2	6.5	24	0	37	20.5	41
	C3	24	4.5	0	0	51.5	40
	C4	1.5	28.5	24	8.5	42.5	94.5
	C5	26	1.5	0	0	26	23.5
Group C	C6	6.5	14.5	2	9	12.5	4
SART3	C7	18	33	9	0	473	254.5
	C8	0.5	3.5	2.5	0.5	5	2
	C9	94	27	0	0	29	35.5
	C10	17	1	0.5	0	18.5	0.5
	C11	132	16.5	0	2	73	86.5
	C12	5	0	0	0	0	0

Study	Patient ID	AFP$_{357}$		AFP403		HIVenv$_{584}$		CMVpp65$_{328}$	
		Pre	Post	Pre	Post	Pre	Post	Pre	Post
	D1	1.5	43.5	0	24.5	4.5	0	192	105
	D2	2	0.5	3.5	1	7	2	28.5	14
	D3	14.5	288	2	21	3	0.5	3.5	3.5
	D4	0	1	0	0	2	0	146	33.5
	D5	0	0	0	0	1.5	8	1.5	17
	D6	0	1.5	1.5	0.5	0.5	2	0.5	0.5
Group D	D7	0	2	0	1	0	1	11.5	16
AFP	D8	0.5	0	0	0	0	1.5	0	0
	D9	0	12.5	0	13	0	1.5	38.5	68
	D10	5	5	1.5	1	0	5	22	21.5
	D11	0	2.5	0	0	0	0	10	10
	D12	0	19.5	2.5	3	0	0	123.5	121
	D13	0.5	0	4.5	0	3	0	21	13.5
	D14	0	4.5	0	4.5	0	0	101.5	86
	D15	3.5	10.5	0	11	0	0	138	364.5

Study	Patient ID	MRP3$_{765}$		HIVenv$_{584}$		CMVpp65$_{328}$	
		Pre	Post	Pre	Post	Pre	Post
	E1	0.5	11	0	3	9	23
	E2	1.5	1.5	0	0	12	34
	E3	1	3	0	0	28	25.5
	E4	0.5	0	0	0	61	36
	E5	0	0	0	0.5	12.5	190
Group E	E6	1	13	0	0	49	76
MRP3	E7	0	17.5	0	0	254.5	99
	E8	ND	ND	ND	ND	ND	ND
	E9	2	11.5	0	0	26.5	13
	E10	0	7.5	0	0	1	0
	E11	1	3.5	0	0	230.5	606
	E12	0	20	0	0	5.5	11.5

Immune responses before and after peptide vaccination. The experiments were performed in duplicate. Mean numbers of immunospots per 300,000 PBMCs are shown.

Supplementary Table 4

Peptide-specific TCR sequences

Donor	TCR	Specificity	Number of cells	TRAV	TRBV
A2	A2.57	$hTERT_{461}$	13	8-3*02	19*01
	A2.80	$hTERT_{461}$	1	24*01	11-2*01
	A2.112	$hTERT_{461}$	56	26-1*02	7-6*01
D3	D3.2	AFP_{357}	66	29/DV5*01	9*01
	D3.14	AFP_{357}	1	8-3*01	5-6*01
	D3.16	AFP_{357}	4	34*01	4-2*01
A2	A2-CMV	$CMVpp65_{328}$	6	24*01	9*01
X	CMV-TCR*	$CMVpp65_{328}$	–	24*01	7-3*01

*This TCR was used in the cytotoxicity assay as a control TCR

Supplementary Table 5

Antibodies for flow cytometry

Antigen	Reactivity	clone	Fluorochrome	Manufacturer	Catalog#
CCR7	human	3D12	FITC	Invitrogen	11-1979-42
CD3	human	SK7	APC-H7	BD biosciences	560176
CD45RA	human	HI100	PerCP-Cy5.5	Invitrogen	45-0458-42
CD8α	human	RPA-T8	APC	BD biosciences	561421
CD8α	human	RPA-T8	PerCP-Cy5.5	BD biosciences	560662
CD8α	human	SFCI21Thy2D3	FITC	Beckman Coulter	6603861
CTLA-4	human	BNI3	PE-CF594	BD biosciences	562742
PD-1	human	MIH4	APC	BD biosciences	558694

Supplementary Note – Analysis of scRNAseq data

++++++Cell ranger platform and pipelines+++

Ran cell ranger count with
200820_1249-1-1_20JU07_Sample1_scRNA_S1_L001_R1_001.fastq
200820_1249-1-1_20JU07_Sample1_scRNA_S1_L001_R2_001.fastq
And
200820_1250-1-1_20JU07_Sample2_scRNA_S3_L001_R1_001.fastq
200820_1250-1-1_20JU07_Sample2_scRNA_S3_L001_R2_001.fastq
With a reference of
refdata-cellranger-GRCh38-3.0.0.tar.gz (downloaded from 10x Genomics)

Ran cell ranger aggr to combine Y1 and Y5 data.
After these step we generated .cloupe files from each run.

Ran cell ranger vdj with
200820_1249-2-1_20JU07_Sample1_scTCR_S2_L001_R1_001.fastq
200820_1249-2-1_20JU07_Sample1_scTCR_S2_L001_R2_001.fastq
And
200820_1250-2-1_20JU07_Sample2_scTCR_S4_L001_R1_001.fastq
200820_1250-2-1_20JU07_Sample2_scTCR_S4_L001_R2_001.fastq
With a reference of
refdata-cellranger-vdj-GRCh38-alts-ensembl-5.0.0. (obtained from 10x Genomics)
After these steps we generated .vloupe files

++++++Loupe browser platform++
Linked .vloupe file to .cloupe file on Loupe browser then sought for the known TCR sequences below;

TCR	TRAV	TRAJ	CDR3α	TRBV	TRBJ	TRBD	CDR3β
A2.57	8-3*02	***	AVGALNTGGFKTIF	19*01	***	***	ASSTAWGQGFLNQPQHF
A2.80	24*01	***	AFQTGANNLFF	11-2*01	***	***	ASSPYNEQFF
A2.112	26-1*02	***	IVRVALPYQGAQKLVF	7-6*01	***	***	ASSWGTGGNQPQHF
D3.2	29/DV5*01	***	AASAYSGAGSYQLT	9*01	***	***	ASSVEGQSPTGELF
D3.14	8-3*01	***	AVGVHYGGATNKLI	5-6*01	***	***	ASSPRDSGELF
D3.16	34*01	***	GADGRSNSGYALN	4-2*01	***	***	ASSQDYDPYGYT
A2.CMV	24*01	***	APTTGGKLI	9*01	***	***	ASSVGQGAYTEAF

*** some information are hidden

Identification criteria was TRAV/J match with CDR3a or/and TRBV/D/J match with CDR3b.

Extracted CD3E expression log >0 and CD8A expression log > 0 then generated new tSNE plots.

Using the Y1+Y5 aggregated dataset, we exported gene expression data comparing identified known TCR+ cells and other CD3E+CD8A+ cells (source_data_Suppl. Fig. 9). In addition, we chose 18 genes of interest and exported expression data of those comparing each TCR and total CD8 T cells (source_data_Suppl. Fig. 8).

For Y1 and Y5 datasets, we exported gene expression data comparing cluster 2 and the others(source_data_Suppl. Fig. 5–6).

++++++++R software+++
Exported data; peptide_specific, Y1_CL2, and Y5_CL2 (source_data_Suppl. Fig. 5–6) were converted into volcano plots using a graphic package -ggplot.

Exported data; source_data_Suppl. Fig. 8 was visualized as a heatmap.

++++++++++++Cytoscape software++
Exported data; Y1_CL2, and Y5_CL2 were imported into Cytoscape software. Using GeneMANIA add-in database, we generated pathway maps. Log2 fold change values were reflected to the node color and p-values were shown by the node size.
