# Supplementary material for: Peptide vaccine-treated, long-term surviving cancer patients harbor self-renewing tumor-specific CD8+ T cells
Source: Nat Commun. 2022 Jun 3;13:3123. doi: 10.1038/s41467-022-30861-z (PMC9166698; doi:10.1038/s41467-022-30861-z)
Supplement: Supplementary file 3 — Reporting Summary [file 41467_2022_30861_MOESM3_ESM.pdf]

nature research

Corresponding author(s): Eishiro Mizukoshi

Last updated by author(s): Apr 8, 2022

Reporting Summary

Nature Research wishes to improve the reproducibility of the work that we publish. This form provides structure for consistency and transparency in reporting. For further information on Nature Research policies, see our Editorial Policies and the Editorial Policy Checklist.

Statistics

For all statistical analyses, confirm that the following items are present in the figure legend, table legend, main text, or Methods section.

n/a	Confirmed	
☐	☒	The exact sample size (*n*) for each experimental group/condition, given as a discrete number and unit of measurement
☐	☒	A statement on whether measurements were taken from distinct samples or whether the same sample was measured repeatedly
☐	☒	The statistical test(s) used AND whether they are one- or two-sided<br>*Only common tests should be described solely by name; describe more complex techniques in the Methods section.*
☐	☒	A description of all covariates tested
☐	☒	A description of any assumptions or corrections, such as tests of normality and adjustment for multiple comparisons
☐	☒	A full description of the statistical parameters including central tendency (e.g. means) or other basic estimates (e.g. regression coefficient) AND variation (e.g. standard deviation) or associated estimates of uncertainty (e.g. confidence intervals)
☐	☒	For null hypothesis testing, the test statistic (e.g. *F*, *t*, *r*) with confidence intervals, effect sizes, degrees of freedom and *P* value noted<br>*Give P values as exact values whenever suitable.*
☒	☐	For Bayesian analysis, information on the choice of priors and Markov chain Monte Carlo settings
☒	☐	For hierarchical and complex designs, identification of the appropriate level for tests and full reporting of outcomes
☐	☒	Estimates of effect sizes (e.g. Cohen's *d*, Pearson's *r*), indicating how they were calculated

Our web collection on statistics for biologists contains articles on many of the points above.

Software and code

Policy information about availability of computer code

Data collection	For flow cytometry analyses, we used BD FACSDiva software ver. 6.1.2 for FACSAria II to obtain the raw data and to export FCS files. BD FACSDiva is not free application but widely used with BD FACS flow cytometry/cell sorters.

Data analysis	To carry out the statistical analyses and to output graphs (Fig. 1c-d, Fig. 2c-f, Fig. 2h-I, Fig. 3, Fig. 4b-c, e, g and Table 1-5), we used Prism (v_9.0.0 & v_9.1.0) (Graph Pad). The adopted statistical methods are described in each figure legend. In Table 1-5, we used Fisher's exact test to evaluate the relationship between CTL responses and CMV responses. To analyze the FCS files, we used FlowJo ver. 10.7.1 (BD) to obtain frequencies of the certain population and generated figure components (Fig. 2a-b, g, Fig5a-b).<br>For RNAseq/TCRseq analysis, the raw data were processed using 10x Cell Ranger ver. 4.0 (10x Genomics) to output .cloupe files and .vloupe files. We linked TCR sequence data and transcriptome data on Loupe browser 4.0 (10x genomics) and generated the tSNE plots with various information and exported the gene expression data. To make the volcano graphs and heatmap (Fig. 5g-h, Suppl. Fig.8-9), we used R software. To draw the pathway maps, we used Cytoscape ver. 3.8.2 software with GeneMANIA database (Suppl. Fig. 5-6).<br><br>GraphPad Prism (GraphPad) is a commercial application, however, is widely used and reliable. Trial version of Prism is available at https://www.graphpad.com.<br>FlowJo (BD) is a commercial application. Free trial version is available at https://www.flowjo.com/solutions/flowjo/free-trial.<br>10x Cell ranger and Loupe browser are available for free at https://support.10xgenomics.com/single-cell-gene-expression/software/overview/welcome.<br>R software is available at https://www.r-project.org.<br>Cytoscape is available at https://cytoscape.org.<br>GeneMANIA add-in is installable in the application.<br><br>We provide more detailed information as Supplementary information – Analysis of scRNAseq data.

For manuscripts utilizing custom algorithms or software that are central to the research but not yet described in published literature, software must be made available to editors and reviewers. We strongly encourage code deposition in a community repository (e.g. GitHub). See the Nature Research guidelines for submitting code & software for further information.

Data

Policy information about availability of data

All manuscripts must include a data availability statement. This statement should provide the following information, where applicable:

- Accession codes, unique identifiers, or web links for publicly available datasets
- A list of figures that have associated raw data
- A description of any restrictions on data availability

Source data are provided with this paper. The RNAseq data generated in this study have been deposited in the DNA data bank of Japan (DDBJ) under the bioproject number PRJDB11756 [https://ddbj.nig.ac.jp/resource/bioproject/PRJDB11756]. The raw RNAseq data are available in the depository. The processed data are available in the Source data file.

Field-specific reporting

Please select the one below that is the best fit for your research. If you are not sure, read the appropriate sections before making your selection.

☒ Life sciences ☐ Behavioural & social sciences ☐ Ecological, evolutionary & environmental sciences

For a reference copy of the document with all sections, see nature.com/documents/nr-reporting-summary-flat.pdf

Life sciences study design

All studies must disclose on these points even when the disclosure is negative.

Sample size	We analyzed the datasets from five P1 trials testing peptide vaccine treatment for HCC patients. The primary endpoints of those studies were to evaluate safety and efficacy of the peptide vaccines, therefore the sample sizes were set as minimal (12-15 cases/trial).<br><br>The sample sizes were determined to examine safety and immunogeneicity of the peptide vaccines. Each trial recruited 10-15 participants, since primary/secondary endopoints did not include the efficacy of the treatments and minimal numbers were required.
Data exclusions	We did exclude some individuals to conduct flow cytometry and ELISpot assays due to sample availability -for example, patients' death or difficulty in following-up in the up to 10-year periods- as clearly shown in Fig. 3.
Replication	The cell line-based experiments were performed twice (Fig. 4b-e). Each trial showed the faithful result.<br>Because we tested patient's specimens at the specific time-points, it was impossible to carry out the replication of the most experiments, instead we performed each experiment in duplicate (Fig.3).
Randomization	Because of the study design, we did not take the randomization.<br>The primary purpose was to test safety of the vaccines, excluding covariates was not required.
Blinding	We did not take group allocation in the series of P-I study because all of them were single-arm trials.

Reporting for specific materials, systems and methods

We require information from authors about some types of materials, experimental systems and methods used in many studies. Here, indicate whether each material, system or method listed is relevant to your study. If you are not sure if a list item applies to your research, read the appropriate section before selecting a response.

Materials & experimental systems

n/a	Involved in the study
☐	☒ Antibodies
☐	☒ Eukaryotic cell lines
☒	☐ Palaeontology and archaeology
☒	☐ Animals and other organisms
☐	☒ Human research participants
☐	☒ Clinical data
☒	☐ Dual use research of concern

Methods

n/a	Involved in the study
☒	☐ ChIP-seq
☐	☒ Flow cytometry
☒	☐ MRI-based neuroimaging

Antibodies

Antibodies used

Antigen,Reactivity,clone,Fluorochrome,Manufacturer,Catalog #,Lot#, running dilution
CCR7,human,3D12,FITC,Invitrogen,11-1979-42,2162087, 1:100
CD3,human,SK7,APC-H7,BD biosciences,560176,1021392, 1:100
CD45RA,human,HI100,PerCP Cy5.5,Invitrogen,45-0458-42,1993639, 1:100
CD8α,human,RPA-T8,APC,BD biosciences,561421,7221992, 1:100
CD8α,human,RPA-T8,PerCP Cy5.5,BD biosciences,560662,9290508, 1:100
CD8α,human, SFCI21Thy2D3,FITC,Beckman,6603861,7241050F, 1:50
CTLA-4,human,BNI3,PE-CF594,BD biosciences,562742,9002841, 1:100
PD-1,human,MIH4,APC,BD biosciences,558694,9037635, 1:100

Validation

All information including reactivity, references and validation data are well described at each website of the manufacturer as below;

CCR7,human,3D12,FITC,Invitrogen,11-1979-42,2162087
> https://www.thermofisher.com/order/genome-database/dataSheetPdf?producttype=antibody&productsubtype=antibody_primary&productId=11-1979-42&version=146CD3,human,SK7,APC-H7,BD biosciences,560176,1021392

CD45RA,human,HI100,PerCP Cy5.5,Invitrogen,45-0458-42,1993639
> https://www.thermofisher.com/order/genome-database/dataSheetPdf?producttype=antibody&productsubtype=antibody_primary&productId=45-0458-42&version=146

CD8α,human,RPA-T8,APC,BD biosciences,561421,7221992
> https://www.bdbiosciences.com/us/reagents/research/antibodies-buffers/immunology-reagents/anti-non-human-primate-antibodies/cell-surface-antigens/apc-mouse-anti-human-cd8-rpa-t8/p/561421+&cd=1&hl=ja&ct=clnk&gl=jp&client=safari

CD8α,human,RPA-T8,PerCP Cy5.5,BD biosciences,560662,9290508
> https://www.bdbiosciences.com/us/p/560662+&cd=2&hl=ja&ct=clnk&gl=jp&client=safari

CD8α,human, SFCI21Thy2D3,FITC,Beckman,6603861,7241050F
> https://www.beckman.com/reagents/coulter-flow-cytometry/antibodies-and-kits/single-color-antibodies/cd8/6603861

CTLA-4,human,BNI3,PE-CF594,BD biosciences,562742,9002841
> https://www.bdbiosciences.com/us/p/562742+&cd=1&hl=ja&ct=clnk&gl=jp&client=safari

PD-1,human,MIH4,APC,BD biosciences,558694,9037635
> https://www.bdbiosciences.com/us/p/558694+&cd=1&hl=ja&ct=clnk&gl=jp&client=safari

Eukaryotic cell lines

Policy information about cell lines

Cell line source(s)

C1R-A24: Originally established at Kumamoto University School of Medicine, transferred to Kanazawa University, and currently maintained and stored in our laboratory.

HepG2: Established at Wistar Institute. We purchased HepG2 from ATCC and maintained in our laboratory.

Phoenix-A: Established at Stanford University (Dr. Nolan) and currently maintained at ATCC. Under Dr. Nolan's MTA policy, our cells were kindly provided by Dr. Kishi at Toyama University.

K-562: lymphoblast cell line purchased from ATCC and maintained in our laboratory.

Authentication

We did not conduct any procedures of authentication for the cell lines above.

Mycoplasma contamination

The cell lines used were negative for Mycoplasma contamination.

Commonly misidentified lines
(See ICLAC register)

None.

Human research participants

Policy information about studies involving human research participants

Population characteristics

We analyzed the participants of the following P1 clinical trials;
A: Clinical trial of hTERT-derived peptide vaccine for hepatocellular carcinoma
B: Clinical trial of chondroitin-glucuronate C5-epimerase-derived peptide vaccine for hepatocellular carcinoma
C: Clinical trial of human homologue of Prp24p-derived peptide vaccine for hepatocellular carcinoma
D: Clinical trial of AFP-derived peptide vaccine for hepatocellular carcinoma
E: Clinical trial of hepatic arterial infusion chemotherapy with multidrug resistance-associated protein 3-derived peptide for hepatocellular carcinoma

Each clinical trial had certain population characteristics following the protocols.
Common characteristics;
≥20 years of age, no gender restriction, HLA-A24 positive (HLA-A DNA typing, BML, Japan), and histologically or radiologically diagnosed with primary HCC in accordance with the American Association for the Study of Liver Diseases guidelines for management of HCC.

Distinct characteristics between the trials;
A, B, C: Very early stage or early stage of HCC (BCLC staging) who had already been treated with radio frequency thermal ablation therapy to achieve cure (prophylaxis purpose).
D: Advanced stage of HCC (BCLC staging) with no option of standard therapeutics (therapeutic purpose).
E: Intermediate to advanced stage of HCC (BCLC staging). Combination therapy of MRP3 derived-peptide vaccine and 5FU/CDDP hepatic arterial infusion chemotherapy (therapeutic purpose).

Patients demographics and clinical characteristics (also shown in Supplementary Figure 2)
Trial,Age Median,Age Range,Male:Female,LC/non-LC/ND,BCLC 0/A/B/C/D
A,63,52-74,10:4,12/2/0,7/6/1/0/0
B,73,49-79,8:4,5/4/3,7/5/0/0/0
C,70,62-83,9:3,5/7/0,5/6/1/0/0
D,73,55–81,9:6,13/2/0,0/0/8/7/0
E,67,58-74,10:2,10/2/0,0/0/7/5/0

Recruitment

In all of the five clinical studies, the participants were recruited through an open recruitment process. We consider there was no impact of the selection bias on the results described in the manuscript.

Ethics oversight

The study protocols were all approved by Medical Ethics Committee of Kanazawa University.

Note that full information on the approval of the study protocol must also be provided in the manuscript.

Clinical data

Policy information about clinical studies
All manuscripts should comply with the ICMJE guidelines for publication of clinical research and a completed CONSORT checklist must be included with all submissions.

Clinical trial registration

We registered the clinical trials at UMIN Clinical Trials Registry (https://www.umin.ac.jp/ctr/).
UMIN000003511
UMIN000004540
UMIN000005677
UMIN000003514
UMIN000005678

Study protocol

Information are available at the websites below;

https://upload.umin.ac.jp/cgi-open-bin/ctr_e/ctr_view.cgi?recptno=R000004255
https://upload.umin.ac.jp/cgi-open-bin/ctr_e/ctr_view.cgi?recptno=R000005426
https://upload.umin.ac.jp/cgi-open-bin/ctr_e/ctr_view.cgi?recptno=R000006715
https://upload.umin.ac.jp/cgi-open-bin/ctr_e/ctr_view.cgi?recptno=R000004260
https://upload.umin.ac.jp/cgi-open-bin/ctr_e/ctr_view.cgi?recptno=R000006716

The study protocols are submitted together along with the manuscript.

Data collection

Data collection and analyses were done at Kanazawa University.

A UMIN000003511
Recruitment started on 2010/04/26, closed on 2010/9/30.
Data collection was continued until 2020/12/25.

B UMIN000004540
Recruitment started on 2010/11/15, closed on 2011/3/10.
Data collection was continued until 2019/9/11.

	C UMIN000005677<br>Recruitment started on 2011/6/1, closed on 2013/1/18.<br>Data collection was continued until 2020/12/30.<br><br>D UMIN000003514<br>Recruitment started on 2010/4/14, closed on 2012/3/22.<br>Data collection was continued until 2020/12/11.<br><br>E UMIN000005678<br>Recruitment started on 2011/6/1, closed on 2012/7/3.<br>Data collection was continued until 2014/8/13.
Outcomes	A UMIN000003511<br>Safety and feasibility of the peptide vaccine were proved (primary endpoint). Efficacy of immune induction was demonstrated as 50% in terms of TNF production.<br><br>B UMIN000004540<br>Safety and feasibility of the peptide vaccine were proved (primary endpoint). Efficacy of immune induction was demonstrated as 33% in terms of IFNg production.<br><br>C UMIN000005677<br>Safety and feasibility of the peptide vaccine were proved (primary endpoint). Efficacy of immune induction was demonstrated as 33% in terms of IFNg production.<br><br>D UMIN000003514<br>Safety and feasibility of the peptide vaccine were proved (primary endpoint). Efficacy of immune induction was demonstrated as 33% in terms of IFNg production.<br><br>E UMIN000005678<br>Safety and feasibility of the peptide vaccine were proved (primary endpoint). Efficacy of immune induction was demonstrated as 33% in terms of IFNg production.

Flow Cytometry

Plots

Confirm that:

☒ The axis labels state the marker and fluorochrome used (e.g. CD4-FITC).

☒ The axis scales are clearly visible. Include numbers along axes only for bottom left plot of group (a 'group' is an analysis of identical markers).

☒ All plots are contour plots with outliers or pseudocolor plots.

☒ A numerical value for number of cells or percentage (with statistics) is provided.

Methodology

Sample preparation	Patients' whole peripheral blood was taken and immediately added with heparin to avoid coagulation using heparin blood collection tubes (VP-H100K, Terumo Corp., Japan) then subject to ficol density gradient centrifugation. Collected peripheral mononuclear cells (PBMCs) were washed twice and suspended in Cellbanker 1 (Takara bio, Japan) to be ready for frozen stocks of PBMCs.<br>Frozen samples were rapidly thawed in a water bath then washed twice and resuspended in MACS buffer at a cell density of 10 x 10^6/ml or less, which is ready for tetramer/antibody staining for flow cytometry.
Instrument	We used FACSAria II and FACSDiva ver. 6.1.2 for flow cytometry data collection and sorting.
Software	We exported FCS files from FACSDiva then analyzed them on FlowJo ver. 10.7.1 (BD).
Cell population abundance	We analyzed 1,000,000-2,000,000 cells in total per sample.<br>For sorting, we enriched by sorting the rare tetramer+ population (0.005-0.02% as shown in Fig. 6a-b) and mixed them with A2 lymphocytes gated on FSC/SSC and 7AAD–. We provide our strategy as Supplementary information – Sorting strategy for single-cell RNA seq analysis. We did not check the purity after sorting due to the unavailability of the samples left.<br>Based on our experiences, purity post enrichment of such rare populations usually results in low immaculateness around 5-30%.
Gating strategy	Gating strategies are provided as Supplementary information – Gating strategy. We took two types of strategies for analysis (A) and sorting (B). For analysis, we identified CD8+ T cells gated on FSC/SSC gate-in, single-cell gate-in, and CD3e+CD8a+. For frequency comparison, we used the same gating sets on FlowJo and obtained the results as Fig. 3. For sorting, to avoid antibody-related effects during incubation on transcriptome profiles we did not use anti-CD3 antibody. Instead, we adopted 7AAD to exclude dead cells and strict doublet exclusion gates.

☒ Tick this box to confirm that a figure exemplifying the gating strategy is provided in the Supplementary Information.
